# MORN5 Expression during Craniofacial Development and Its Interaction with the BMP and TGFβ Pathways

**DOI:** 10.3389/fphys.2016.00378

**Published:** 2016-08-31

**Authors:** Petra Cela, Marek Hampl, Katherine K. Fu, Michaela Kunova Bosakova, Pavel Krejci, Joy M. Richman, Marcela Buchtova

**Affiliations:** ^1^Institute of Animal Physiology and Genetics, v.v.i., Academy of Sciences of the Czech RepublicBrno, Czech Republic; ^2^Department of Animal Physiology and Immunology, Institute of Experimental Biology, Masaryk UniversityBrno, Czech Republic; ^3^Life Sciences Institute, University of British ColumbiaVancouver, BC, Canada; ^4^Department of Biology, Faculty of Medicine, Masaryk UniversityBrno, Czech Republic; ^5^International Clinical Research Center, St. Anne's University HospitalBrno, Czech Republic

**Keywords:** cleft lip, maxillary prominence, mandibular prominence, frontonasal mass, BMP

## Abstract

MORN5 (MORN repeat containing 5) is encoded by a locus positioned on chromosome 17 in the chicken genome. The MORN motif is found in multiple copies in several proteins including junctophilins or phosphatidylinositol phosphate kinase family and the MORN proteins themselves are found across the animal and plant kingdoms. MORN5 protein has a characteristic punctate pattern in the cytoplasm in immunofluorescence imaging. Previously, *MORN5* was found among differentially expressed genes in a microarray profiling experiment of the chicken embryo head. Here, we provided *in situ* hybridization to analyse, in detail, the *MORN5* expression in chick craniofacial structures. The expression of *MORN5* was first observed at stage HH17-18 (E2.5). *MORN5* expression gradually appeared on either side of the primitive oral cavity, within the maxillary region. At stage HH20 (E3), prominent expression was localized in the mandibular prominences lateral to the midline. From stage HH20 up to HH29 (E6), there was strong expression in restricted regions of the maxillary and mandibular prominences. The frontonasal mass (in the midline of the face) expressed MORN5, starting at HH27 (E5). The expression was concentrated in the corners or globular processes, which will ultimately fuse with the cranial edges of the maxillary prominences. *MORN5* expression was maintained in the fusion zone up to stage HH29. In sections *MORN5* expression was localized preferentially in the mesenchyme. Previously, we examined signals that regulate *MORN5* expression in the face based on a previous microarray study. Here, we validated the array results with *in situ* hybridization and QPCR. *MORN5* was downregulated 24 h after Noggin and/or RA treatment. We also determined that BMP pathway genes are downstream of *MORN5* following siRNA knockdown. Based on these results, we conclude that *MORN5* is both regulated by and required for BMP signaling. The restricted expression of *MORN5* in the lip fusion zone shown here supports the human genetic data in which *MORN5* variants were associated with increased risk of non-syndromic cleft lip with or without cleft palate.

## Introduction

The vertebrate face is formed very early in development from the paired maxillary and mandibular prominences and the single frontonasal mass surrounding the oral cavity. These facial prominences arise during early embryogenesis from interactions between neural crest derived mesenchyme and head ectoderm. The frontonasal mass grows out, contacts and fuses together with the maxillary prominences to form the upper jaw. The midline facial skeleton consisting of the nasal septum, prenasal cartilage and premaxilla are all derived from the frontonasal mass (Richman and Lee, 2003). Craniofacial development is complex process coordinated by a network of transcription factors and signaling molecules (Murray and Schutte, 2004; Chai and Maxson, 2006; Jiang et al., 2006; Brunskill et al., 2014; Kurosaka, 2015; Marcucio et al., 2015; Nimmagadda et al., 2015). Disruption of this tightly controlled cascade can result in clefts where the facial prominences fail to meet and fuse (Leslie and Marazita, 2013).

Cleft lip and/or cleft palate are the most common craniofacial birth defects in humans (Setó-Salvia and Stanier, 2014; Watkins et al., 2014). The majority of clefts appear as isolated or non-syndromic clefts, because they occur in isolation from other developmental abnormalities. The causes of clefting are thought to be multifactorial, including an interaction between genes and the environmental factors (Schutte and Murray, 1999; Dixon et al., 2011; Leslie and Marazita, 2013; Setó-Salvia and Stanier, 2014; Watkins et al., 2014). Identification of genes contributing to clefts formation is important not only for our understanding of facial development, but also for improved prevention and treatment of affected individuals. The chicken embryo is a valuable experimental model to study the signals that control lip fusion. The avian primary palate closely resembles the primary palate in mammals (Abramyan et al., 2015). Moreover, the face can be accessed directly in the living embryo through a window in the shell. The disruption of FGF (Szabo-Rogers et al., 2008), BMP (Ashique et al., 2002), SHH (Hu et al., 2015), and WNT signaling (Geetha-Loganathan et al., 2014) causes a cleft lip in chickens that resembles that of humans.

Previously, a microarray study was performed to profile gene expression in individual chicken facial prominences in stage 18 embryos (Buchtová et al., 2010). From the list of genes that were significantly more highly expressed in the maxillary prominence, we selected *MORN5* (also known as C9orf113, C9orf18 or FLJ46909) for further studies because it was described as a cleft susceptibility gene (Letra et al., 2010). Microarray analysis revealed 24 times higher expression of *MORN5* in the maxillary prominence compared to expression in the frontonasal mass at stage 18, while mandibular prominence showed 10 times higher expression than the frontonasal mass (Buchtová et al., 2010).

Members of the MORN family were named for the presence of multiple MORN motifs (Membrane Occupation and Recognition Nexus). There are five paralogous genes in the *MORN* family (MORN1-5). Limited functional information is available for a subset of MORN genes. *MORN1* has been identified in the parasite *Toxoplasma gondii* and other Apicomplexan protists where it plays role during cell division (Ferguson et al., 2008; Lorestani et al., 2010). Human *MORN2* was found to facilitate phagocytosis-mediated restriction of some bacteria in macrophages (Abnave et al., 2014). Expression of *MORN3* was detected in mouse testis, where it regulates spermatogenesis (Zhang et al., 2015). Finally, *MORN4* promotes axonal degeneration in mouse sensory axons (Bhattacharya et al., 2012).

**Figure 1:**
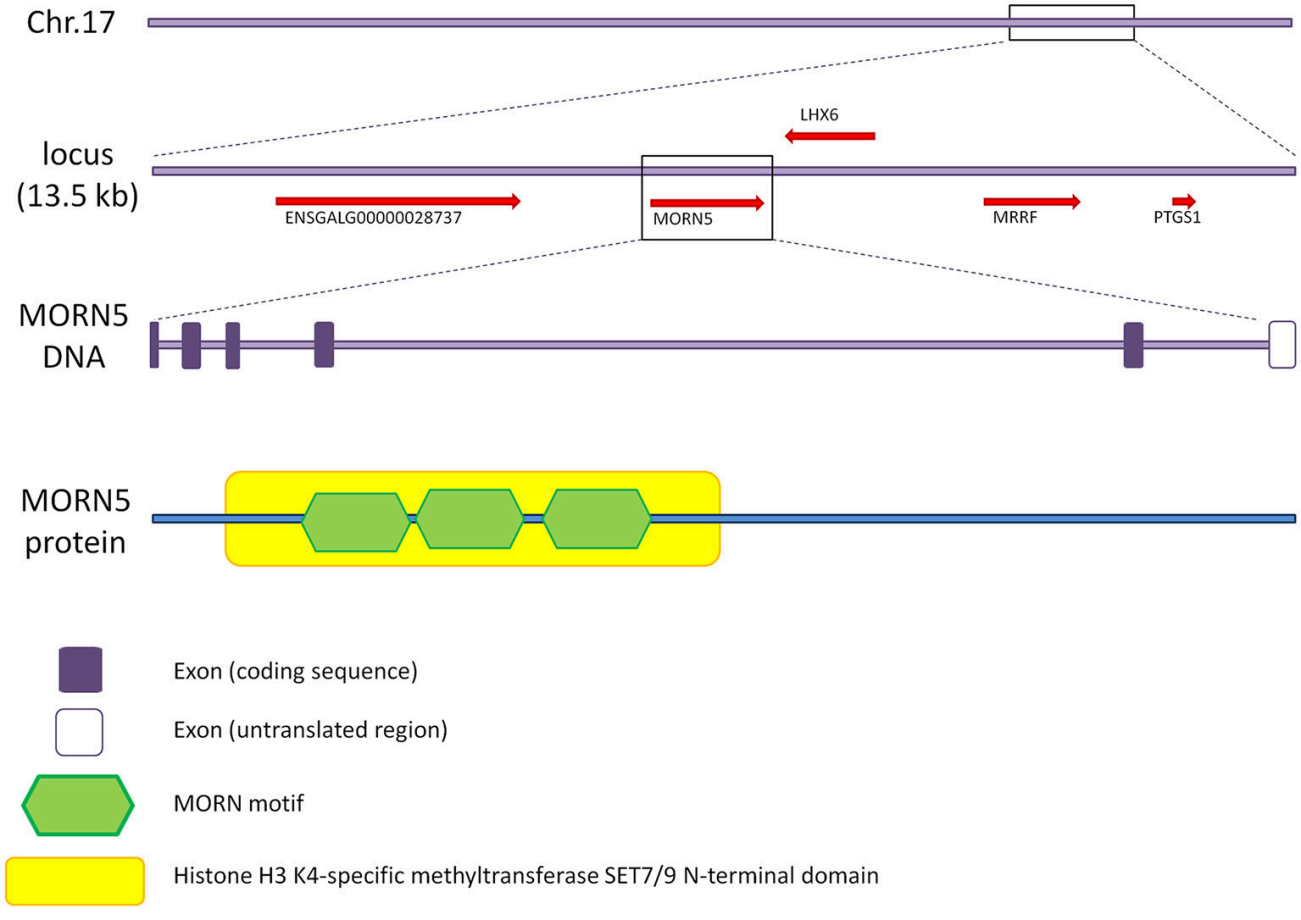
**Gene characteristics of chicken *MORN5* and domain analysis**. *MORN5* gene is located on chromosome 17 of the chicken genome and its length is 13.5 kb. The gene is composed of 6 exons where the last one is non-coding. The open reading frame codes for a protein 172 amino acids in length. The gene contains SSF82185 domain and three MORN motifs.

In chicken, the *MORN5* gene is located on the forward strand of chromosome 17. On the reverse strand, *NDUFA8* and *LHX6* genes are nearby to the *MORN5* gene. The size of the *MORN5* gene is 13.5 kb and there are 6 exons (only 5 exons are coding) with four splice variants. The *MORN5* gene encodes a protein of 172 amino acids, which contains a histone H3 K4-specific methyltransferase SET7/9 N-terminal domain (SSF82185) and three MORN motifs (Figure 1).

As the gene expression pattern or possible function of *MORN5* during development had not been investigated in any animal model, we aim to analyzed chicken *MORN5* expression in embryos and its integration into signaling pathways.

## Materials and methods

### Embryonic material

Fertilized chicken eggs (ISA brown) were obtained from the farm Integra (Žabčice, Czech Republic). Eggs were incubated in a humidified forced air incubator at 37.8°C. Embryos were staged and morphological characteristic were described according to Hamburger and Hamilton (1951). All procedures were conducted following a protocol approved by the Laboratory Animal Science Committee of the Institute of Animal Physiology and Genetics (Liběchov, Czech Republic).

### Section *in situ* hybridization (ISH)

Chicken *MORN5* was obtained as chicken EST clone CHEST ID 543 F09 (Biovalley, France), where the probe sequence was cloned into pBluescript II KS+ vector. The entire region containing the probe sequence flanked by T3 and T7 RNA polymerase sites was amplified using M13 primers (forward primer: 5′-GTA AAA CGA CGG CCA G-3′, reverse primer: 5′-CAG GAA ACA GCT ATG AC-3′). Then, the amplicon was isolated via gel purification (QIAquick Gell Extraction Kit, Qiagen, Germany) and this linearized DNA fragment was used in RNA polymerase reactions. DIG labeled antisense riboprobe was synthesized with T3 RNA polymerase (antisense) or with T7 polymerase (sense controls).

Embryos were fixed in 4% paraformaldehyde (PFA), processed through ethanol and xylene into paraffin, and sectioned for ISH. Hybridization was performed with RNA probe at 60°C overnight as described previously (Holland et al., 1996). Anti-digoxigenin sheep antibody conjugated with alkaline phosphatase (1:2000, Roche, USA) was applied overnight at 4°C. Visualization was achieved by incubation with substrates for alkaline phosphatase (BM Purple AP, Roche, Germany) for several days. Slides were then counterstained with eosin. ISH was carried out on at least three embryos for each stage.

### Embryo manipulations

Embryos were treated with beads soaked in All-trans retinoic acid (RA), Noggin protein, Tris or Dimethyl Sulfoxide (DMSO) as described (Lee et al., 2001). Since DMSO was the solvent for RA, we used DMSO bead as a control for RA treatment and Tris as a control for Noggin treatment. AG1-X2 beads (Bio-Rad Laboratories, Hercules, Canada) of 100 μm in diameter were soaked in RA (cat. No. R2625 Sigma) at a concentration of 1 mg/ml for 1 h as previously described (Lee et al., 2001; Nimmagadda et al., 2015). Noggin proteins were soaked into Affigel blue beads (Bio-Rad Laboratories, Hercules, Canada) of 200 μm diameter for a minimum of 1 h at a concentration of 1 mg/ml (cat. No. 1967-NG, R&D Systems, Minneapolis, USA). Control beads were soaked with DMSO or Tris. Two beads were implanted into the maxillary region on the right side of chicken embryo at stage HH15. For ISH and QPCR, samples were collected 24 h post-bead implantation, embedded into paraffin and processed for ISH.

### Immunofluorescence on slides

Embryos were collected at stage HH24 for MORN5 protein detection. Chicken duodenum was used as a control according to the manufacturer's instruction. Samples were fixed in 4% PFA and processed into paraffin. Following deparaffinization and rehydration, antigen retrieval was carried out using citrate buffer for 1 min at 97°C. Polyclonal antibody to MORN5 (1:50, cat. No. NBP1-91230, Novus Biologicals, USA) was applied overnight at 4°C. The secondary anti-rabbit antibody (1:200, Alexa Fluor 594, cat. No. A-21207) was applied for 30 min at RT. Sections were washed in PBS and coverslipped with Prolong Gold anti-fade reagent containing DAPI (cat. No. P36935, Invitrogen, USA).

### Quantitative RT-PCR

Gene expression of *MORN5* was analyzed on tissues isolated from normal chicken prominences at stage 15, 18, 20, and 26. Moreover, Noggin or RA treated maxillary prominences were dissected 24 h following bead implantation at stage 15. Prominences were pooled from at least 15 embryos to produce one sample and 4 biological replicates were analyzed. Total RNA was extracted using the Mini RNeasy Kit (Qiagen, Germany) according to the manufacturer's instructions. The total RNA concentration and purity of each sample were assessed by spectrophotometry using a NanoDrop1000 (Thermo Scientific, Waltham, USA). First-strand cDNA was synthesized using the SuperScript Vilo cDNA synthesis Kit (cat. No. 11754050, Thermo Fisher, USA). The qPCR was performed in 10 μl final reaction volumes containing the one-step master mix (no AmpErase UNG, cat. No. 4324018, Applied Biosystems, Carlsbad, USA) mixed with *MORN5* (TaqMan Assays, Assay ID: AJKAKYV, context sequence: TTCCTGAGAAATGCAGACGATGAGG, FAM-MGB, Applied Biosystems, Austin, USA) on LightCycler® 96 (Roche, Manheim, Germany) with preheating at 95°C/10 min, followed by 40 cycles of 95°C/15 s and 60°C/1 min. Gene expression levels were calculated using ΔΔCT method with normalization against the *HPRT1* level (TaqMan Assays, Assay ID: Gg033338900_m1, context sequence: TTGAATCATATCTGTGTGATCAGTG, FAM-MGB, Applied Biosystems, Austin, USA), which was used as the housekeeping gene. Means of 3 technical replicates were generated for each of 3 biological replicates and these values were used for statistical analysis. All procedures were repeated in at least three independent experiments.

### Transfection with MORN5 plasmids in cell cultures

The expression vector containing C-terminally FLAG-tagged human MORN5 was obtained from OriGene (Rockville, MD). HEK293T cells were obtained from ATCC (Manassas, VA) and propagated in DMEM media (Sigma-Aldrich, St. Louis, MO) with 10% fetal bovine serum, 1% Pen/Strep and 1% l-glutamin (Invitrogen, Carlsbad, CA). Cells were transfected using FUGENE6 reagent according to manufacturer's protocol (Promega).

HEK293T cells grown on glass coverslips were fixed with 4% PFA (RT/15 min), permeabilized with 0.1%Triton-X100 in PBS (RT/5 min), and incubated with the following antibodies at 4°C overnight: MORN5 (1:100, cat. No. NBP1-91230, Novus Biologicals), FLAG (1:200, cat. No. F1804, Sigma-Aldrich). The secondary antibody AlexaFluor 488 (1:500; cat. No. A21206, Life Technologies) or AlexaFluor 594 (1:500; cat. No. A21203, Life Technologies) were used. Coverslips were mounted into DAPI-containing Mowiol. Images were taken on an LSM700 laser scanning microscope with acquisition done using ZEN Black 2012 software (Zeiss, Jenna, Germany).

### siRNA targeting gMORN5 in chicken embryos

Silencer Select custom designed siRNA (gMORN5, cat. No. 4399666, Ambion, Austin, USA) was mixed with FUGENE 6 (Roche, Mannheim, Germany), and then was injected into the maxillary prominence of chicken embryos. Negative siRNA (Silencer select negative control No.1 siRNA, cat. No. 4390843, Ambion, Austin, USA) was used as a control. The first injection of siRNA was performed at stage HH20 and the second one after 24 h about stage HH24. One day later after the second injection, embryos had reached stage HH28 and maxillary prominences were dissected for RNA isolation. Tissues were dissected from 5 embryos to form one sample and three biological samples were used for treated embryos (MORN5 siRNA) as well as for control (Silencer select negative control No.1 siRNA) embryos.

### PCR arrays

Total RNA was extracted from siRNA treated maxillary prominences using the Mini RNeasy Kit (Qiagen, Germany) according to the manufacturer's instructions. First-strand cDNA was synthesized using the SuperScript Vilo cDNA synthesis Kit (cat. No. 11754050, Thermo Fisher, USA). Downregulation of *MORN5* expression after injection was first confirmed using qPCR before further processing for PCR Array analysis.

Custom made Chick-bone plates (KRD, Czech Republic) were used for analysis of BMP pathway genes. The PCR arrays were performed in 12 μl final reaction volumes containing SYBR Premix Ex Taq II (cat. No RR0821A, Takara, Japan) on LightCycler® 96 (Roche, Manheim, Germany) with preheating at 95°C/30 min, followed by 45 cycles of 95°C/5 s, 60°C/20 s and 72°C/15 s. Data were statistically evaluated by ΔΔCT method with normalization against *HPRT1* levels. In each PCR array plate, there were three technical replicates for 24 genes, and 2 technical replicates for an additional 13 genes.

### Statistical analysis

All results were expressed as means ± standard deviations (SD) of three samples for each treatment and were compared by unpaired two-tailed Student's *t*-test for qPCR and PCR Array. Differences were considered to be significant at *p* < 0.05.

## Results

### Spatiotemporal gene expression pattern of *MORN5* in facial prominences

**Figure 2:**
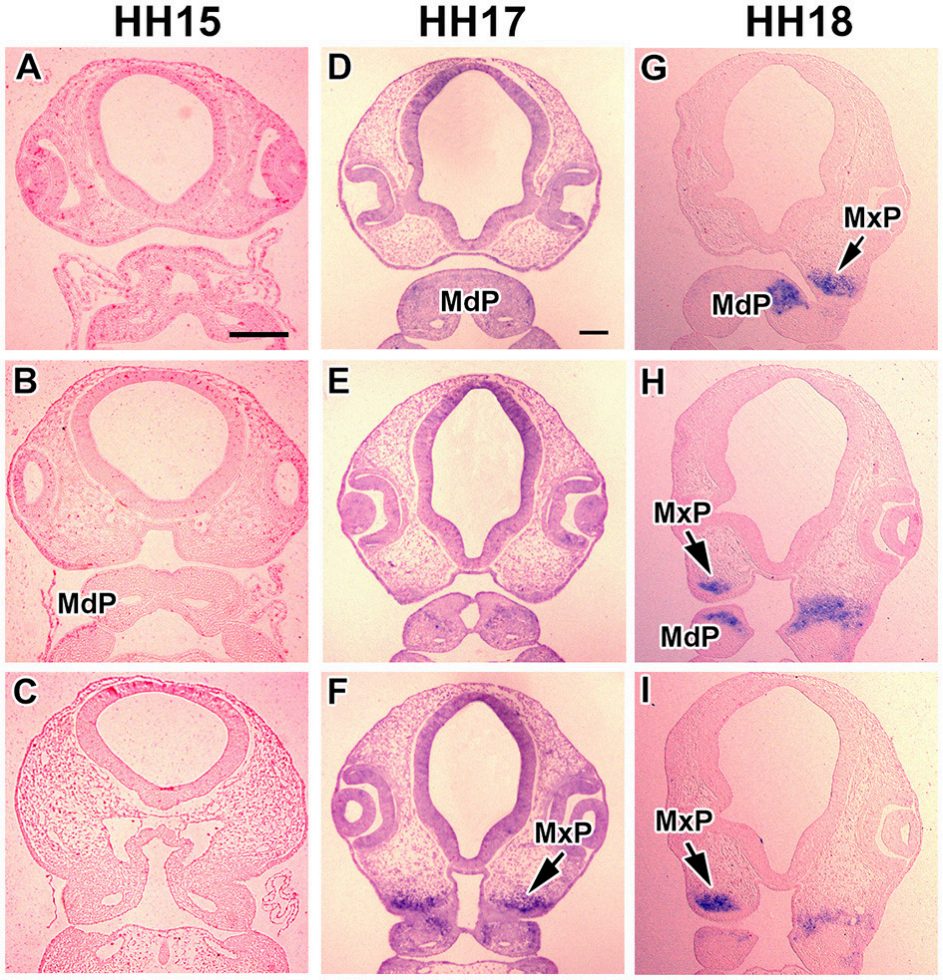
**Gene expression of *MORN5* in early chicken face. (A–C)** Frontal sections of chicken face at stage HH15. **(D–F)** Frontal sections of chicken face at stage 17. **(D,E)** In the ventral part of maxillary and mandibular prominences, there was no expression. **(F)** *MORN5* expression gradually appeared dorsally in caudal part of maxillary region. **(G–I)** Frontal sections of chicken face at stage HH18. *MORN5* expression was strong in maxilla and also in central part of each mandibular prominence **(G)**. MdP, mandibular prominence; MxP, maxillary prominence. Scale bars = 100 μm.

**Figure 3:**
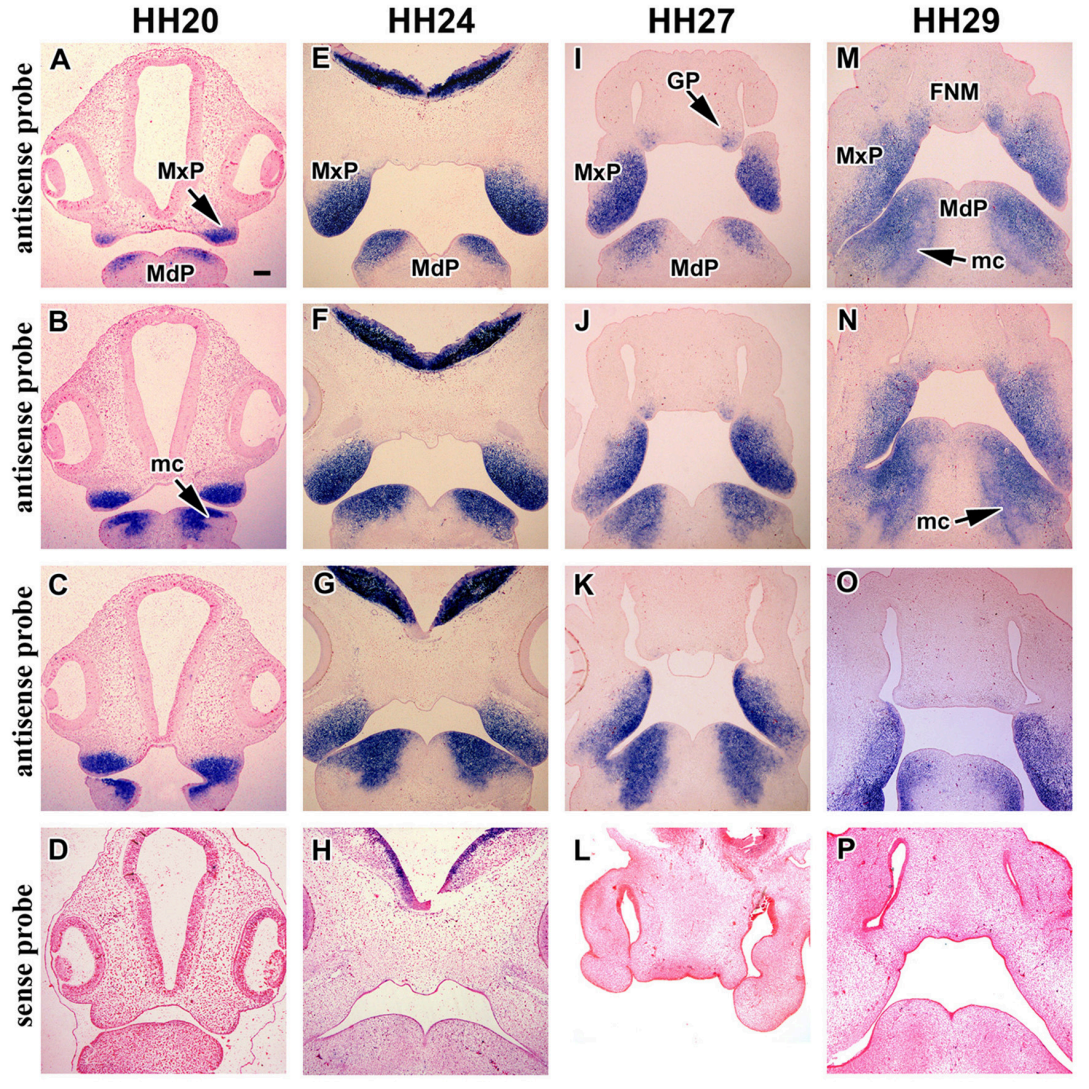
**Gene expression of *MORN5* in later stages of chicken embryo. (A–C)** ISH analysis in frontal sections of chicken head at stage HH20. There was strong expression in the maxillary prominence. The expression appeared in the cranial part in each mandibular prominence and it continued in dorsal direction. **(B)** No expression was observed in mesenchymal condensations and close to the fusion region of mandibular prominences. **(E–G)** *MORN5* expression in frontal section of chicken head at stage HH24. *MORN5* expression was strong in the mesenchyme of maxillary prominences. *MORN5* expression was localized in the dorsal part of mandibular prominence, but not in mesenchymal condensation and close to the midline. **(I–K)** Frontal sections of chicken head at stage HH27 showed prominent expression in the maxillary prominence. There was weak expression in the globular process. Prominent expression was observed in rostral part of maxillary prominence and also in the mandibular prominence with the exception of midline. **(M–O)** Frontal sections of chicken head at stage 29 where beak is evident. *MORN5* expression was localized in rostral part of maxilla and in fusion region. In the mandibular prominence, there was strong expression but not in the midline. **(D,H,L,P)** ISH analysis using sense probe. FNM, frontonasal mass; GP, globular process; mc, mesenchymal condensation; MdP, mandibular prominence; MxP, maxillary prominence. Scale bars = 100 μm.

First, we analyzed spatiotemporal expression pattern of *MORN5* in individual prominences of chicken face. Facial prominences begin to form during early embryonic development. *In situ* hybridization showed no expression in chicken face at Hamburger-Hamilton (HH) stage 15 (50–55 h of incubation, Figures 2A–C) which is shortly after neural crest cells have entered the face. Later at stage HH17 (52-64 h of incubation, Figures 2D–F), *MORN5* expression appeared in the caudal part of the presumptive maxillary mesenchyme close to the maxillo-mandibular cleft. At stage HH18, the bulge of the maxillary prominence contained high levels of *MORN5* transcripts (65–69 h of incubation, Figures 2G–I). Expression was also detected in the dorsal (oral side) part of the mandibular prominences close to the maxillo-mandibular cleft (Figures 2G,H). At stage HH20 (70–72 h of incubation), there continued to be restricted expression in caudal and medial domains within the maxillary prominences (Figures 3A–C). In the mandibular prominences, there was expression in the cranial mesenchyme on either side of the midline groove (Figures 3A–O) with the exceptions of mesenchymal condensations of Meckel's cartilage (Figures 3B,C). At stage HH24 (4 days of incubation), maxillary prominence enlarged and strong *MORN5* expression was present throughout the mesenchyme (Figures 3E–G). Mandibular expression was similar to stage HH20 (Figures 2E–G). Thus, *MORN5* is expressed in a restricted pattern in neural crest-derived mesenchyme but not in epithelium. Sense probe did not show signal in the maxillary prominence (Figures 3D,H,L,P).

### *MORN5* expression in the lip fusion zone at later stages

**Figure 4:**
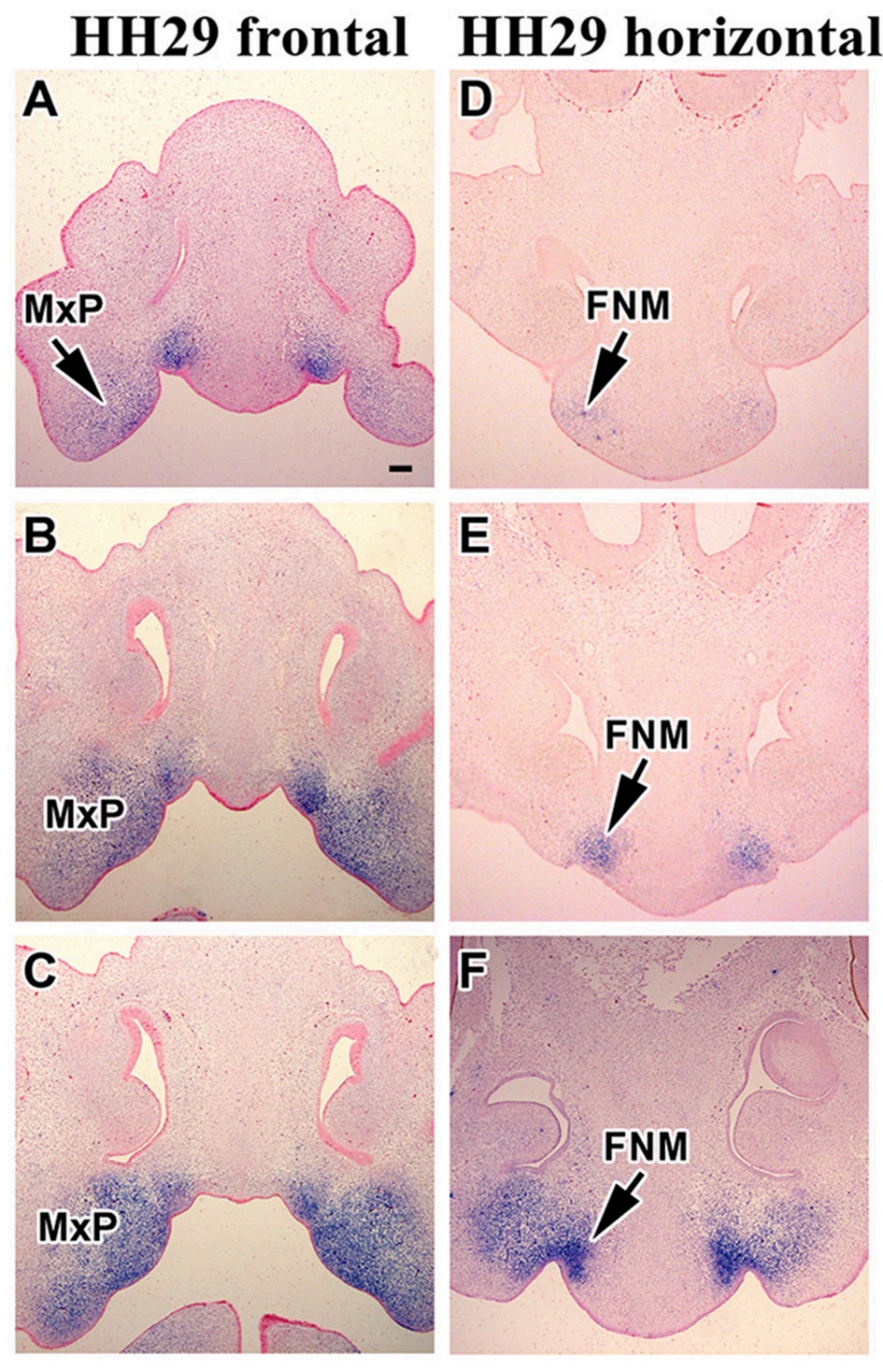
***MORN5* expression in the fusion region at stage 29. (A–C)** Frontal sections of chicken head showed strong expression in the maxilla and in the area where edges of the maxillary prominences grow together with medial nasal prominence. **(D–F)** Horizontal sections of chicken head. **(E)** Region of fusion had prominent *MORN5* expression. **(F)** More caudal section (other sample). Scale bars = 100 μm.

The next critical phase of facial morphogenesis is the fusion of the lip. Between stage HH27–29, the cranial-medial edges of the maxillary prominences meet the lateral corners of medial nasal prominences (globular processes) and fuse (Abramyan et al., 2015). At stage HH27 (5 days of incubation), *MORN5* expression was observed for the first time in the corners of the frontonasal mass (globular processes, Figures 3I,J). Expression in the maxillary prominences was high in the rostral-medial corner just where fusion with the globular processes will take place. There continued to be expression in the mandibular prominences similar to stage 24 (Figures 3I–K). At stage HH29 (6 days of incubation), *MORN5* expression was located in the region of lip fusion (Figures 3M,N) as well as in the mandible. This is the first stage where expression of *MORN5* in Meckel's cartilage was detected (Figure 3M). Further confirmation of the restricted expression in the lip fusion zone is shown in other embryos cut in the frontal (Figures 4A–C) or transverse plane at stage HH29 (Figures 4A–F). Note that mesenchymal bridging has taken place by stage HH29, unifying the domains of expression of *MORN5* in the globular processes and maxillary prominences (Figures 4B,C).

**Figure 5:**
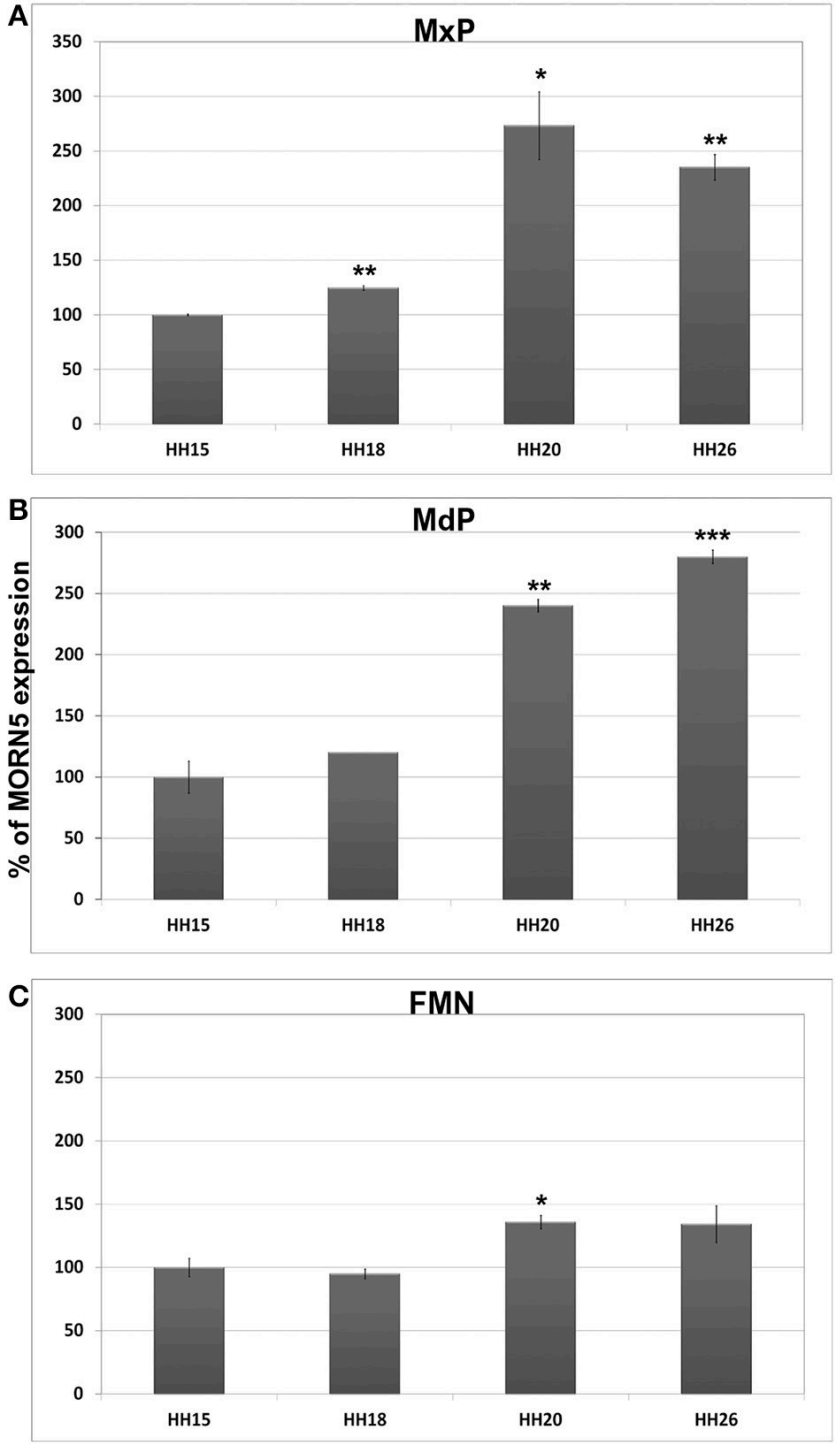
**QPCR analysis of *MORN5* expression during face development. (A)** In the maxillary prominence, there was most prominent expression at stage HH20. All analyzed stages showed statistically significant overexpression in comparison to stage HH15. **(B)** In the mandibular prominence, we observed significant expression at stage HH20 and 26. **(C)** Very low *MORN5* expression was detected in the frontonasal mass at stage HH15 and 18, but at stage HH20 was *MORN5* expression significantly increased. FNM, frontonasal mass; MdP, mandibular prominence; MxP, maxillary prominence. *t*-test; ^***^p < 0.001, ^**^0.001 < *p* < 0.01, ^*^p < 0.05.

To quantify the relative levels of expression between the stages of development, we performed QPCR for evaluation of *MORN5* expression level in each prominence at four different stages (HH15, 18, 20, 26). Since stage HH15, we did not observe any expression of *MORN5* by ISH, this level of expression was chosen as the reference value for ΔΔCt analysis for individual prominences. In the maxillary prominence, *MORN5* expression gradually increased during development with the peak level seen at stage HH20 (Figure 5A). In the mandibular prominence, we observed significantly increased expression at stage HH20 and 26 compared to stage HH15 embryos (Figure 5B). In the frontonasal mass, *MORN5* expression is very low except of the globular processes we were surprised to see a statistically significant increase in expression of stage HH20 embryos (Figure 5C). In the section of *in situ* experiments, we could not detect *MORN5* at stage HH20 (data not shown) therefore sensitivity of QPCR is greater than *in situ* hybridization. By stage HH27, there is expression of *MORN5* in the *in situ* experiments; however, QPCR data did not pick up a significant expression level in stage HH26 embryos (Figure 5C). Some of the variability may be due to the dissection process and whether the globular process was included in all the samples. We did not compare expression levels between the facial prominences due to the experimental design.

### MORN5 protein expression in the face

**Figure 6:**
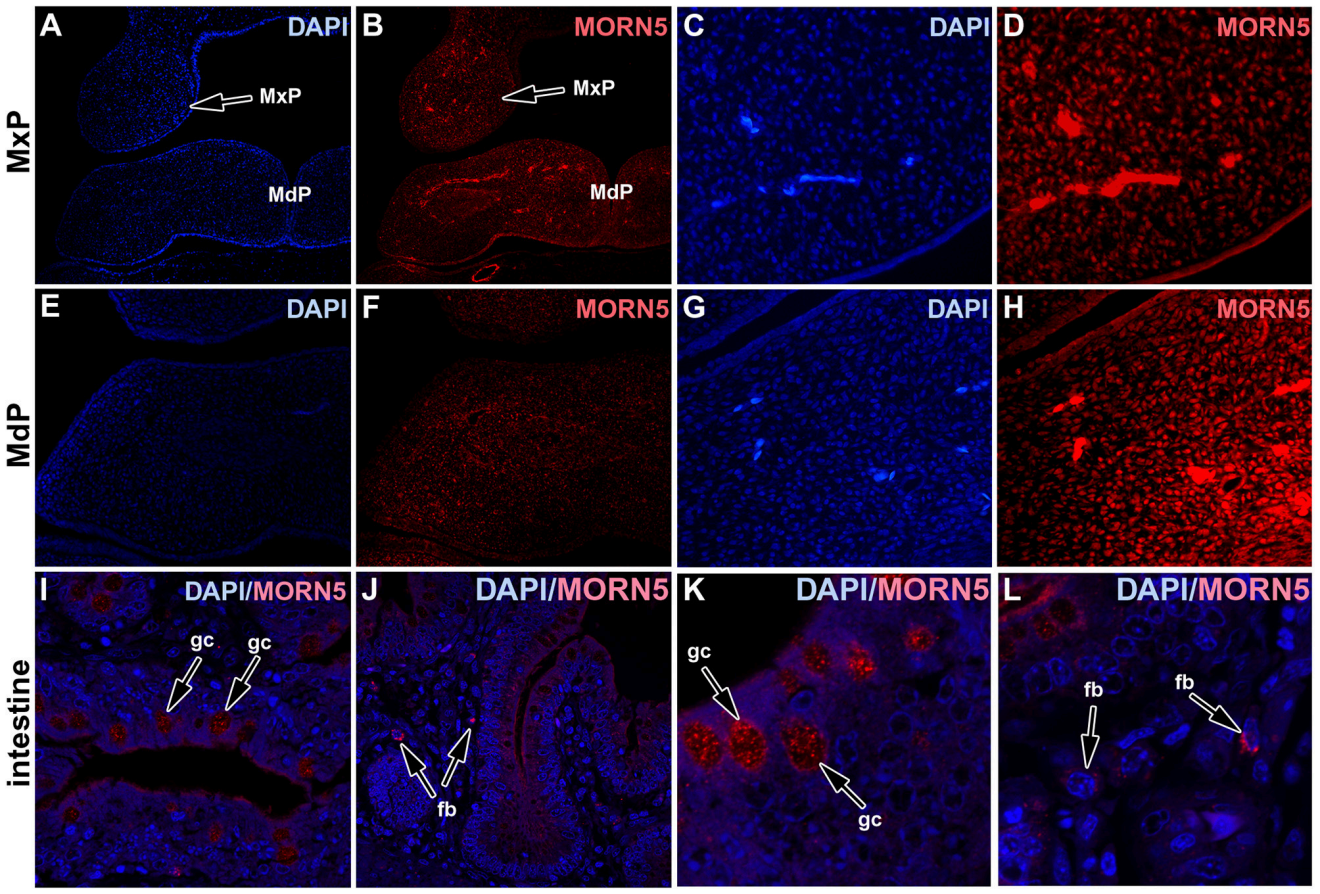
**MORN5 protein detected by immunofluorescent labeling. (B,D)** MORN5 protein was localized in the maxillary prominences at stage HH24. **(F,H)** In the mandibular prominence, there is very low signal in the mesenchyme. MORN5 expression showed dispersed pattern of distribution. **(A,C,E,G)** DAPI staining. **(I–L)** Chicken intestine was used as a positive control with strong positivity in Goblet cells (gc), in the apical parts of enterocytes and also spotted expression in fibroblasts (fb). MdP, mandibular prominence; MxP, maxillary prominence.

To correlate MORN5 protein distribution with *MORN5* gene expression, we performed immunofluorescence staining. MORN5 protein was localized in developing chicken face at stage HH24, with the most prominent expression in individual cells in the maxillary and mandibular prominences (Figures 6A–H). Thus, only a subset of cells expressing *MORN5* RNA expresses the protein. In positive control (adult chicken intestine), there was expected signal in Goblet cells, in the apical parts of enterocytes and in fibroblasts of the lamina propria (Figures 6I–L).

**Figure 7:**
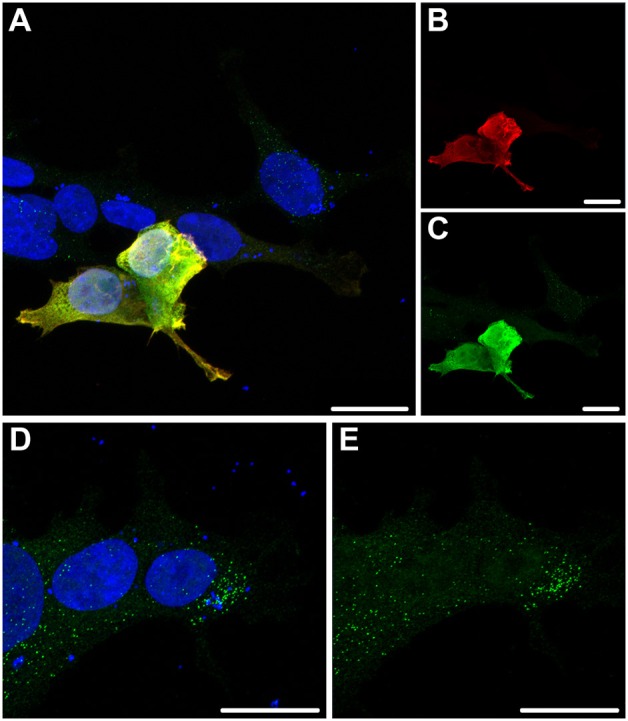
**Cytosolic spotted pattern of MORN5 expression in HEK293T cells. (A–C)** HEK293T cells were transfected with FLAG-tagged MORN5-expressing vector and immunostained using both FLAG and MORN5 antibodies. Cells expressing transgenic MORN5 were also MORN5-positive pointing to the specificity of the antibody. **(D,E)** In addition, a cytosolic spotted pattern of MORN5 expression was present in non-transfected cells. Scale bars = 100 μm.

The specificity of the MORN5 antibody was also confirmed in HEK293T cells transfected with a MORN5-FLAG plasmid (Figures 7A–C). The staining of MORN5 and FLAG antibodies overlapped (Figures 7A–C). Similar to tissue section data, exogenous MORN5 protein was found in the cytoplasm in a punctate pattern (Figures 7D,E).

### Downregulation of *MORN5* after noggin a retinoic acid treatment

**Figure 8:**
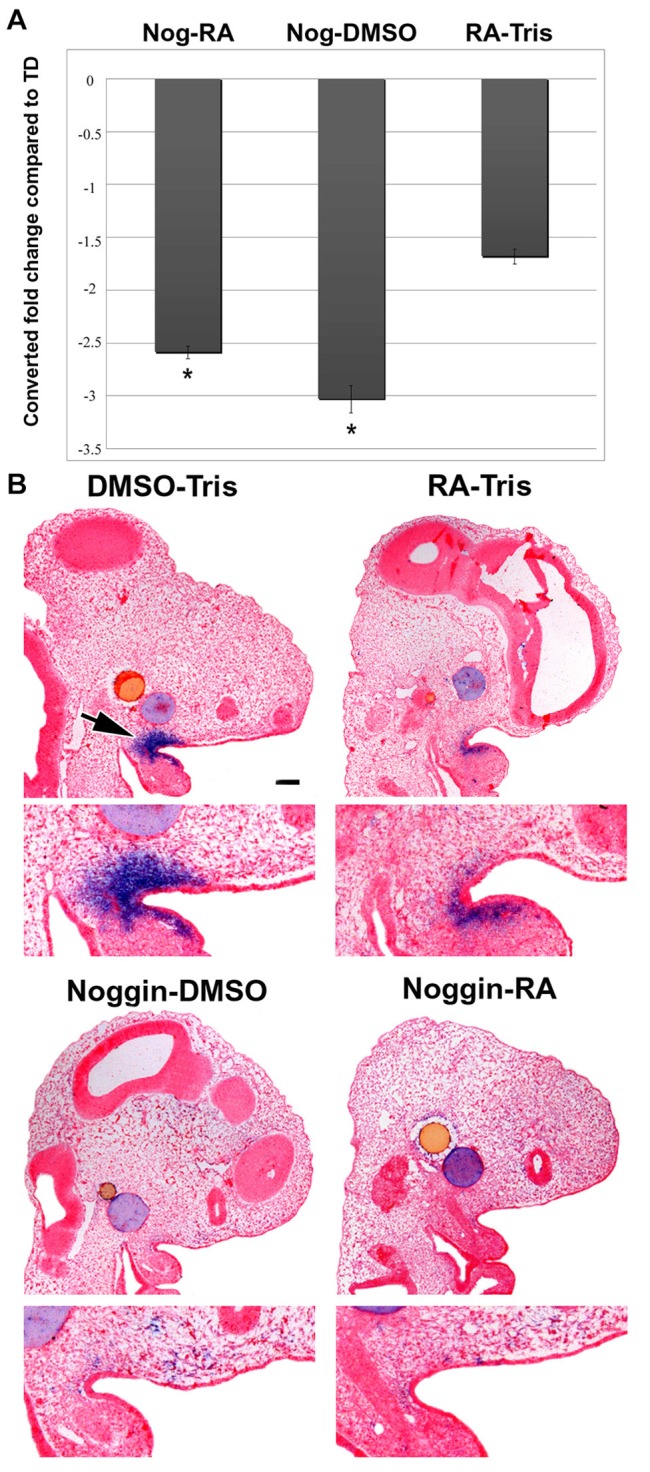
**QPCR analysis and ISH of *MORN5* expression after bead implantation. (A)** QPCR analysis showed 1.68 times downregulation after RA-Tris, 3.03 after Noggin-DMSO and 2.59 after Noggin-RA treatment in comparison to control DMSO-Tris. **(B)** Control embryos implanted with beads soaked in DMSO-Tris had strong expression in maxillary region and maxilla-mandibular cleft. In RA-Tris treated embryos was very weak *MORN5* expression. After Noggin-DMSO treatment, expression was rapidly decreased. No expression of MORN5 was observed after Noggin-RA treatment. Nog, Noggin; RA, Retinoic acid; TD, Tris-DMSO. Scale bars = 100 μm. *t*-test; ^*^p < 0.05.

Our study uncovered high levels of *MORN5* expression in normal chicken embryos, however a previous study from our group discovered that *MORN5* was downregulated in an experimental paradigm involving beads implanted into the chicken face (Nimmagadda et al., 2015). Beads soaked in the bone morphogenetic protein antagonist, Noggin and retinoic acid (RA) synergistically induced transformation of the maxillary prominence into the frontonasal mass (Lee et al., 2001). The tissues from embryos induced to form this duplicated beak were profiled using microarrays. A significant downregulation of *MORN5* expression was observed in all the treatment groups compared to controls treated with DMSO-Tris beads (-3.77-fold Noggin-DMSO treatment, -3.68-fold Noggin-RA, -2-fold after RA-Tris treatment) (Nimmagadda et al., 2015). We wanted to follow up this findings since it appeared that the RA and BMP pathways were upstream regulators of *MORN5* and that possibly *MORN5* was one of a set of genes mediating the beak duplication phenotype. First, we validated the array results using QPCR on maxillary tissues collected from treated and control embryos. We found a significant downregulation of *MORN5* after Nogin-RA and Noggin-DMSO treatment compared to Tris-DMSO controls (Figure 8A). Next, we asked whether there were any spatial differences in *MORN5* expression induced by the bead implants using *in situ* hybridization. Control embryos implanted with beads soaked in DMSO-Tris showed strong expression in the maxillary region and maxillo-mandibular cleft (Figure 8B). In contrast, no expression was observed in the maxillary prominence of Noggin-RA or Noggin-DMSO treated embryos. Interestingly, there was residual expression of *MORN5* observed in embryos treated with RA-Tris located just under the epithelium of maxilla-mandibular cleft (Figure 8B).

### Downregulation of *MORN5* by siRNA altered gene expression of BMP and TGFβ pathways members

**Figure 9:**
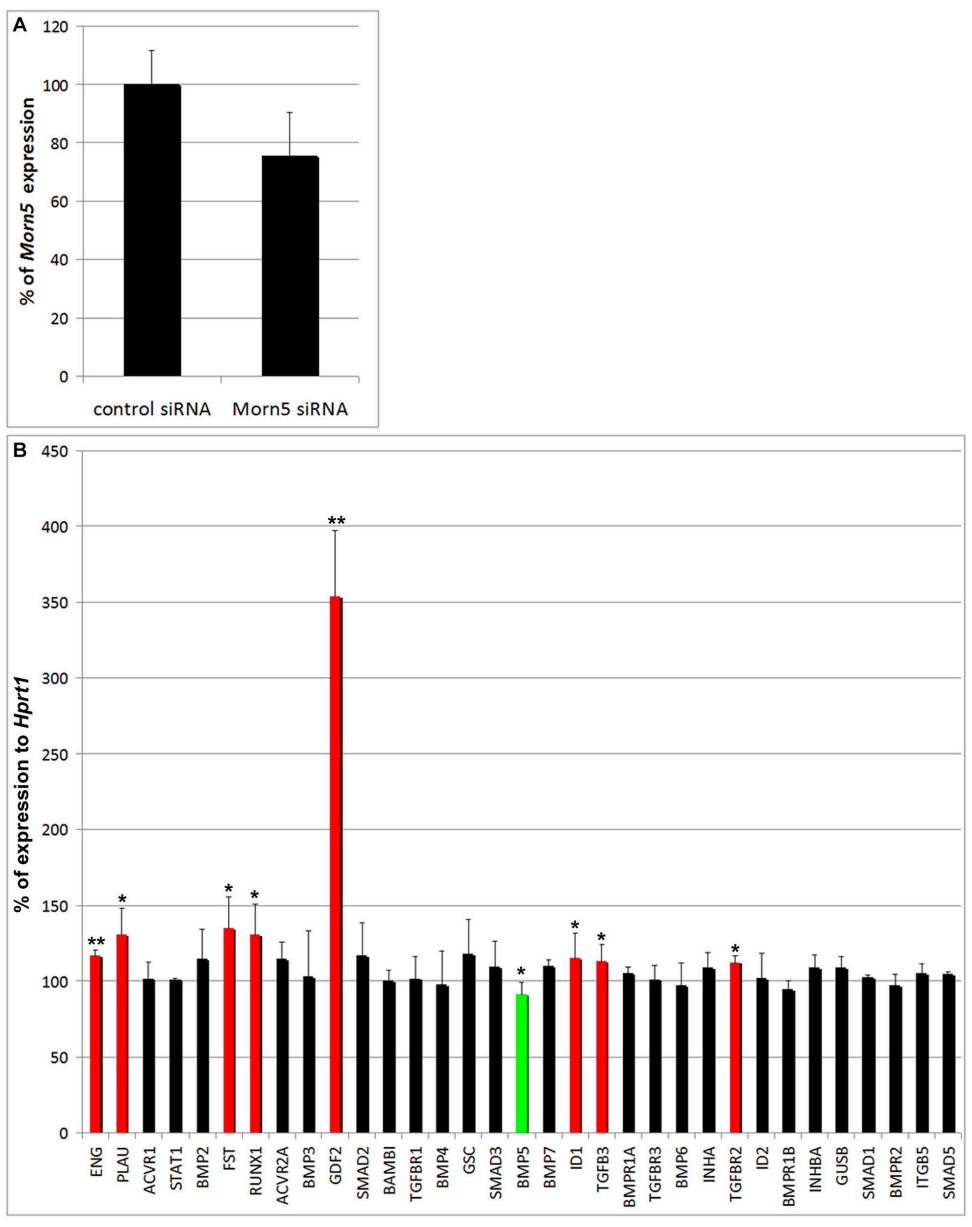
**BMP pathway gene expression after *MORN5* downregulation. (A)** Downregulation of *MORN5* expression after siRNA treatment. **(B)** *MORN5* downregulation caused significant increase in *GDF2, ENG, TGFBR2, TGFB3, PLAU, FST, RUNX1*, and *ID1* expression. Statistically significant downregulation was observed only in case of *BMP5*. *t*-test; ^**^0.001 < *p* < 0.01, ^*^p < 0.05.

We had discovered that BMP activity was required for *MORN5* expression but next wanted to investigate the genes that might be downstream of *MORN5*. As the first group of potential targets, we studied genes that are known to be in the BMP pathway. *MORN5* expression in the maxillary prominence was downregulated to 75% of control levels following transfection with siRNA (2 rounds of transfection: at stage 20 and 24; Figure 9A). We used a PCR array that included 34 genes specific for the BMP pathway with *HPRT1* acting as the reference control gene (Table S1).

Eight genes showed a statistically significant increase in their expression caused by partial *MORN5* silencing (Figure 9B). These included *ENG* (Endoglin), *Gdf2* (Growth differentiation factor 2, also BMP9)*, PLAU* (plasminogen activator, urokinase)*, FST* (Follistatin)*, Runx1* (Runt-related transcription factor 1)*, ID1* (Inhibitor of DNA binding 1), *TGF*β*R2* (Transforming growth factor beta receptor 2) and *TGF*β*3* (Figure 9B). The most striking increase was seen with *GDF2* (increased 3.5-fold). Statistically significant downregulation was observed only in the case of *BMP5* (Figure 9B). It is interesting that *MORN5* normally represses *ID1*, a transcription factor that positively regulates BMP signaling. Although levels of *ID1* were increased, which should imply higher BMP signaling, there is also decreased expression of the *BMP5* ligand. It is likely that cytoplasmic *MORN5* indirectly regulates the expression of these genes and that further work is needed to determine the intermediate mediators of BMP and TGFβ signaling affected by *MORN5*.

## Discussion

Here, we found spatially and temporally restricted expression of *MORN5* in the face area during embryonic development suggesting its role in patterning of the maxillary prominences. Moreover, there was expression in the globular processes of frontonasal mass just before their fusion with the maxillary prominences. Previously, a human genetics study found that *MORN5* was associated with non-syndromic cleft lip with or without cleft palate (NSCLP) (Letra et al., 2010). We are the first to document expression of *MORN5* in the relevant parts of the face undergoing lip fusion. In addition, there is strong expression on the medial sides of the maxillary prominences, the sites where palatal shelves will arise. While the chicken has a naturally cleft palate, it is interesting that *MORN5* is expressed in the intermediate stages of palatal shelf formation. Based on previous microarray studies carried out on the chicken face, *MORN5* came up twice, once as a maxillary enriched gene (Buchtová et al., 2010) and second as a differentially expressed gene following Noggin and RA bead implants (Nimmagadda et al., 2015). Taken together, the human genetic and chicken data suggest that *MORN5* is an important maxillary patterning and possibly lip fusion gene. Thus, it would be worthwhile targeting *MORN5* using mouse models and to include this gene in human NSLCP studies.

Complex signaling interactions coordinate the outgrowth of facial prominences to form the adult face. Some of factors have been previously identified by whole genome expression screens or by candidate gene mapping. The BMP signaling pathway regulates many cellular processes of craniofacial development and it is necessary for mesenchymal outgrowth of facial prominences. The expression of BMPs in chicken face was found at the time prior and during lip fusion (Ashique et al., 2002). *BMP4* transcripts were previously detected in the epithelium of the globular processes of frontonasal mass and *MORN5* expression underlays the same area however entirely in the mesenchyme. Also in the maxillary and mandibular prominences, epithelial *BMP4* expression was described in parallel areas to mesenchymal *MORN5*. Furthermore, *BMP2* and *BMP7* were previously detected in the mesenchyme of both facial prominences. Maxillary and mandibular prominences express also several downstream target of BMP signaling. *MSX1* is strongly expressed in the maxillary prominence but in slightly different pattern than *MORN5* in the mandible (Shigetani et al., 2000; Fuchs et al., 2010). There are additional transcription factors that appear to overlap with *MORN5* specifically in the frontonasal mass globular processes and maxillary prominences, *TBX22, DLX5*, and *MSX2* (Higashihori et al., 2010). These transcription factors may regulate expression of *MORN5*. Interestingly, in the microarray study on beak duplicated embryos, *TBX22* was upregulated following Noggin-RA treatment and *TBX22* acts as a transcriptional repressor. It will be necessary to analyze whether *MORN5* is a target of *TBX22* which is a known clefting gene in humans (Kantaputra et al., 2011). It is interesting to note that the gene *LHX6* which is located 3′ to *MORN5* on the opposite strand was reported to be highly expressed in the chicken face (Washbourne and Cox, 2006). Expression of *LHX6* begins in the maxillo-mandibular cleft at stage 18 similar to *MORN5* (Washbourne and Cox, 2006). There is also striking similarity of expression of *LHX6* in the globular processes and medial maxillary prominences at stage 27. This suggests that the two genes may share some common enhancers that drive expression in particular regions of the face.

*MORN5* knockdown revealed indirect roles for this gene in controlling the expression of BMP and TGFB signaling pathways. Since we have shown MORN5 is a cytoplasmic protein it is unlikely that it is directly involved in regulating gene transcription. Further biochemical studies are needed to determine the exact function of MORN5 in the cell. Nevertheless, the RNA changes we observed suggest a subset of genes are dependent on *MORN5* for their expression. We did not study the TGFB pathway in our bead implantation studies; therefore, the PCR array data extended our original findings on *MORN5* function. The most highly upregulated gene, *GDF2*, is known to associate with Endoglin (Castonguay et al., 2011) a glycoprotein located on cell surfaces that serves as a co-receptor for members of the Transforming growth factor-β superfamily (Cheifetz et al., 1992). This suggests that activity of TGFβ family is normally repressed by MORN5. Other changes such as the increase in the antagonist *FST (Follistatin)* may indicate that MORN5 operates in another way to regulate TGF signaling. MORN5 may normally repress this antagonist, which binds members of the TGFβ superfamily with a particular focus on activin (Lambert-Messerlian et al., 2007). We have shown that *FST* does not induce skeletal changes in the palate as compared to Noggin (Celá et al., 2016) therefore *FST* regulation by *MORN5* may serve different functions outside of facial morphogenesis. Several other genes known to be in the TGFB pathway and essential for mouse palate development were upregulated including *RUNX1* (Yamashiro et al., 2002), TGFβ3 (Cui et al., 1998) and *ID1* (Rice et al., 2005). In summary, we discovered that *MORN5* is involved in TGFB signaling at all levels. In conclusion, BMP signaling is required for *MORN5* expression and reduction of *MORN5* derepresses several genes in the BMP and TGFβ signaling pathways. Furthermore, *MORN5* has two potential roles in facial patterning, to specify maxillary identity and to regulate lip fusion that warrant further study in animal models.

## Author contributions

MB, JR, and PC conceived the study. PC, MH, KF, MK conducted the experiments. PK provided intellectual contribution. PC, JR, and MB wrote the manuscript. All authors reviewed and approved the final manuscript.

### Conflict of interest statement

The authors declare that the research was conducted in the absence of any commercial or financial relationships that could be construed as a potential conflict of interest.
