# Supplementary material for: MORN5 Expression during Craniofacial Development and Its Interaction with the BMP and TGFβ Pathways
Source: Front Physiol. 2016 Aug 31;7:378. doi: 10.3389/fphys.2016.00378 (PMC5005375; doi:10.3389/fphys.2016.00378)
Supplement: Supplementary file 1 [file Table1.DOC]

**Table S1:** List of statistically significant genes on PCR Array

|  | **Gene expression on PCR Array (%)** | **P value** | **Specific gene function** |
| --- | --- | --- | --- |
| Membrane receptors | | | |
| ENG | 116 | 0,005491533 | - serves as a co-receptor for members of the transforming growth factor-β superfamily (Cheifetz et al., 1992) |
| Transcription factors | | | |
| RUNX1 | 130 | 0,047726606 | - involved in development of facial bones and teeth and was associated with palate formation (Yamashiro et al, 2002). |
| Transcription regulators | | | |
| ID1 | 121 | 0,043796718 | - promotes cell cycle progression and help facilitate cell migration (Ling et al., 2014)   Id1 transcripts were detected in both  the palatal mesenchyme and epithe-  lium  Id1 transcripts were detected in both  the palatal mesenchyme and epithe-  lium   - is expressed in epithelium and also in mesenchyme during palatal development (Rice et al., 2005) - expression of Id is upregulated by BMPs (Rice et al., 2005) |
| Transforming growth factors | | | |
| TGFB3 | 118 | 0,017603253 | - it is expressed in epithelium on the corners of palatal shelves in mouse (Fitzpatrick et al., 1990) and also in human (Abbott et al., 1998). - mouse deficient in *Tgfß3* showed palatal cleft (Proetzel et al., 1995). - chicken palatal shelves fused after TGFβ3 treatment *in vitro* (Sun et al., 1998). |
| TGFBR2 | 112 | 0,025908363 | - this receptor was detected in epithelium of palatal shelves in mouse (Cui et al., 1998) - specific deletion of Tgfβr2 in palatal epithelial cells lead to cleft of soft palate, submucous cleft or failure in fusion among primary and secondary palate (Xu et al., 2006) |
| Bone morphogenetic proteins | | | |
| GDF2 | 353 | 0,00616762 | - directly and with high affinity bound to Endoglin (Castonguay et al., 2011) - together with BMP-2 promotes chondrogenic differentiation of human multipotential mesenchymal cell (Majumdar et al., 2001). |
| BMP5 | 91 | 0,022571105 | - required for skeletal patterning during embryonic development and also for bone response and remodeling to mechanical stimulation at specific anatomic sites in the skeleton. (Ho et al., 2008) |
| Activin antagonist | | | |
| FST | 135 | 0,030331081 | - binds members of the TGF-β superfamily with a particular focus on activin. It is also implicated to be important for palatal development (Lambert-Messerlian et al., 2007). - important role during palatal closure (Levi et al., 2006). - antagonizes Tgfß3-induced epithelial-mesenchymal transition in vitro (Nogai et al., 2008). |
| Enzymes | | | |
| PLAU | 131 | 0,028569465 | - encodes a serine protease involved in degradation of the extracellular matrix and it was described to be present in developing  palate just before its fusion in mouse (Melnick et al., 1998) |

**References**

Rice R, Thesleff I, Rice DP (2005) Regulation of Twist, Snail, and Id1 is conserved between the developing murine palate and tooth. Dev Dyn. *234(1):*28-35.

Majumdar MK, Wang E, Morris EA (2001) BMP-2 and BMP-9 promotes chondrogenic differentiation of human multipotential mesenchymal cells and overcomes the inhibitory effect of IL-1. J Cell Physiol. *189(3):*275-84.

Levi G1, Mantero S, Barbieri O, Cantatore D, Paleari L, Beverdam A, Genova F, Robert B, Merlo GR (2006) Msx1 and Dlx5 act independently in development of craniofacial skeleton, but converge on the regulation of Bmp signaling in palate formation. Mech Dev. *123(1):*3-16.

Cui XM, Warburton D, Zhao J, Crowe DL, Shuler CF (1998) Immunohistochemical localization of TGF-beta type II receptor and TGF-beta3 during palatogenesis in vivo and in vitro. Int J Dev Biol. *42(6):*817-20.

Fitzpatrick DR, Denhez F, Kondaiah P, Akhurst RJ (1990) Differential expression of TGF beta isoforms in murine palatogenesis. Development. *109:* 585-95.

Abbott BD, Probst MR, Perdew GH, Buckalew AR (1998) AH receptor, ARNT, glucocorticoid receptor, EGF receptor, EGF, TGF alpha, TGF beta 1, TGF beta 2, and TGF beta 3 expression in human embryonic palate, and effects of 2,3,7,8-tetrachlorodibenzo-pdioxin (TCDD). Teratology. *58 (2):* 30-43.

Proetzel G, Pawlowski SA, Wiles MV, Yin M, Boivin GP, Howles PN, Ding J, Ferguson MW, Doetschman T (1995) Transforming growth factor-beta 3 is required for secondary palate fusion. Nature Genetics. *11(4):* 409-14.

Sun D, Vanderburg CR, Odierna GS, Hay ED (1998) TGFbeta3 promotes transformation of chicken palate medial edge epithelium to mesenchyme in vitro. Development. *125:* 95-105.

Xu X, Han J, Ito Y, Bringas JR, Urata MM, Chai Y (2006) Cell autonomous requirement for Tgfbr2 in the disappearance of medial edge epithelium during palatal fusion. Dev Biol. *297(1):* 238-48.
